# Editorial: Model-based evaluation of antimicrobial agents in children—volume II

**DOI:** 10.3389/fphar.2023.1272996

**Published:** 2023-08-14

**Authors:** Wei Zhao, John Van Den Anker

**Affiliations:** ^1^ Department of Clinical Pharmacy, Key Laboratory of Chemical Biology (Ministry of Education), School of Pharmaceutical Sciences, Cheeloo College of Medicine, Shandong University, Jinan, China; ^2^ NMPA Key Laboratory for Clinical Research and Evaluation of Innovative Drug, Qilu Hospital of Shandong University, Shandong University, Jinan, China; ^3^ Division of Clinical Pharmacology, Children’s National Hospital, Washington, DC, United States; ^4^ Departments of Pediatrics, Pharmacology & Physiology, Genomics & Precision Medicine, The George Washington University School of Medicine and Health Sciences, Washington, DC, United States; ^5^ Department of Pediatric Pharmacology and Pharmacometrics, University of Basel Children’s Hospital, Basel, Switzerland

**Keywords:** model-based drug development, model-based individualized antimicrobial therapy, children, antimicrobial agents, machine learning

## Introduction

Off-label drug use is very common in neonatal and pediatric patients (Tang B H et al., 2023). The pediatric population shows major changes in the way they can handle drugs due to their continuous growth and development. It is therefore very challenging to determine the most effective and safe way to use drugs in neonates, infants, and children. To improve this situation increasing numbers of scholars are working tirelessly to achieve model-based pediatric drug development as well as individualized treatment.

In *“Model-based evaluation of antimicrobial agents in children—volume II*,*”* the articles focus on studies of model-based drug development of antimicrobial agents in the pediatric population as well as model-based individualized antimicrobial therapy in neonates, infants, children, and adolescents.

## Model-based antimicrobial therapy in severe infectious disease

Model-based evaluation of antimicrobial agents has been achieved in severe infectious diseases including pneumonia, critically illnesses and central nervous system infections in children. For antibiotic therapy, most studies are still happening at the level of pharmacokinetics (PK) and pharmacodynamics (PD). The clinical use of individualized therapy and the promotion of precision dosing software has, until now, only be applied in a few antibiotics. Girdwood et al. emphasized the dilemma between endorsing and implementing precision dosing of *β*-lactam antibiotics in critically ill children without knowing which subgroups would really benefit from precision dosing.

In applying knowledge of model-based PK of antibiotics to clinical practice, some scholars are experimenting by conducting randomized controlled trials. Based on a previously published population pharmacokinetic (PopPK) model, Tian et al. designed a randomized controlled trial of meropenem in pediatric patients with severe pneumonia with the aim of comparing the efficacy and safety of the conventional and model-based dosing regimens of meropenem. Compared to the conventional treatment group (20 mg/kg, q8 h, infusion for 0.5–1 h), the model treatment group was optimized using an extended infusion time of 4 h. From a pharmacokinetic perspective, the extended infusion time contributed to the 70% fT > MIC (the free plasma concentration of meropenem exceeds the minimum inhibitory concentration during 70% of the dosing interval) target, and in terms of clinical effectiveness, this model-based dosing regimen should be evaluated.

However, for some diseases, the first step to achieve model-based dose optimization is still to supplement the basic data for efficacy, safety, and pharmacokinetics. Liu et al. reviewed the efficacy, safety, and pharmacokinetics of intravenous and intraventricular vancomycin for central nervous system infection including meningitis, ventriculitis, and central nervous system (CNS) device-associated infections. They found significant differences in dosages, duration, and concentrations of vancomycin in the cerebrospinal fluid for intravenous and intraventricular vancomycin administration, respectively. The optimal dose for the treatment of CNS remains unclear and needs to be achieved by high-level clinical trials assisted by modeling techniques.

Bayesian techniques play a crucial role in model-based antimicrobial therapy. Bunn et al. described the challenges associated with designing and implementing dose optimization strategies and provided evidence that Bayesian-model informed precision dosing can address these challenges. They also described the practice of optimizing vancomycin dose using a software Lyv (Pumas-AI, Inc., Centreville, VA).

## Model-based evaluation of antimicrobial agents in children: state of the art and future

**FIGURE 1:**
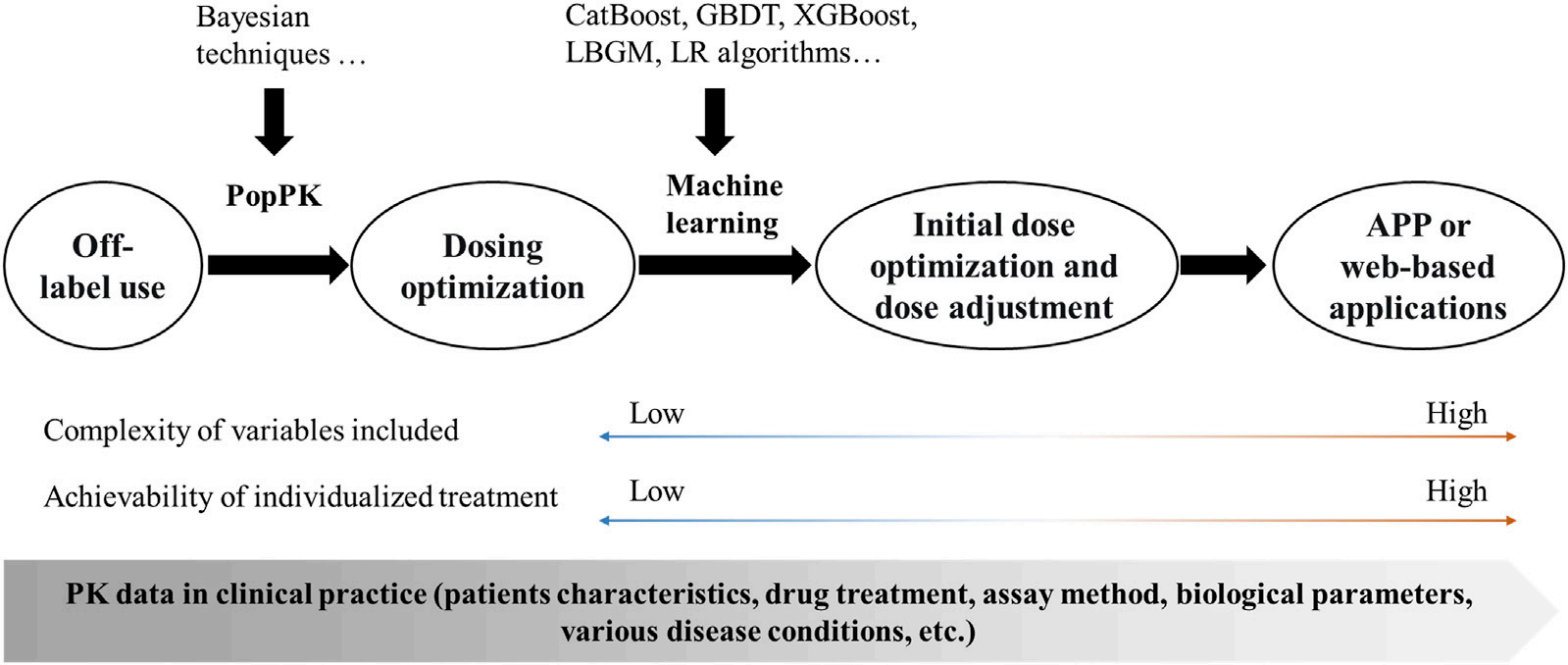
Framework for the implementation of individualized treatment. PopPK, population pharmacokinetics; CatBoost, Categorical boosting; GBDT, Gradient boosting decision tree; XGBoost, Extreme gradient boosting; LBGM, Light gradient boosting machine; LR, Logistic Regression; APP, application; PK, pharmacokinetics.

The PopPK model is a traditional method for achieving dose optimization and has largely improved the phenomenon of off-label drug use in pediatric populations. Despite its widespread use, there are significant problems with this approach: 1) Bayesian estimation based on the PopPK model is not sufficiently accurate and cumbersome; 2) the software often relied upon, such as NONMEM, is limited by the software itself and does not allow for easy application; and 3) traditional models incorporate limited variables and cannot handle complex clinical scenarios. Machine learning is a data-driven technique that can make accurate judgements and predictions about real-world events. It is now widely used in aspects of disease diagnosis (Esteva et al., 2017; Ding et al., 2019), creating many new clinical possibilities and showing great potential in the field of individualized treatment of pediatric patients with antimicrobial agents. Machine learning can handle more complex variable factors and enable initial dose optimization and subsequent dose adjustment, as well as making it easier to convert formulation information into application software or web-based applications for individualized treatment in clinical practice (Figure 1).

By studying six drugs that are primarily eliminated by the kidney (vancomycin, latamoxef, cefepime, azlocillin, ceftazidime, and amoxicillin) as “proof-of-concept” compounds, Tang et al. validated that a combination of PopPK and machine learning approaches could more accurately predict the clearance of drugs for renal elimination in neonates (Tang et al., 2021). In case of vancomycin, steady-state trough concentration (C_0_) and area under the steady-state curve (AUC_0-24_) targets are often used for dose optimization of vancomycin. C_0_-based and AUC_0-24_-based machine learning models of vancomycin have been successfully developed and can achieve individualized initial dose prediction and further optimization based on therapeutic drug monitoring results in neonates by accurately predicting C_0_ and AUC_0-24_ (Tang L et al., 2023).

To summarize, *“Model-based evaluation of antimicrobial agents in children—volume II”* provides current knowledge and future research ideas for model-based drug development and individualized treatment of antimicrobial drugs, in the hope of opening new horizons for the rational use of antimicrobial drugs in children.
